# Unit of Analysis Guide, Part 2: Not Everything That Can Be Counted Counts

**DOI:** 10.1093/asj/sjae124

**Published:** 2024-06-03

**Authors:** Isabella F Churchill, Lucas Gallo, Xi M Zhu, Steven Hanna, Christopher J Coroneos

Researchers often choose study designs that evaluate surgical treatments in a sample of females with measurements of both breasts. Such a design can enhance the study's power, potentially requiring the recruitment of fewer patients.^1^ However, careful consideration is imperative to ascertain the applicability of this design in specific situations and to avoid unit of analysis errors.^2,3^ The correct analysis depends on both the comparison of interest and whether it is reasonable to assume that the outcomes for 2 breasts of the same female are independent. Such an assumptions implies that the clinical and surgical factors are not more similar for 2 breasts of the same female than for breast of 2 different females. In part 2 of this guide, we describe scenarios in which breasts are the unit of analysis, outline statistical assumptions and limitations, and critically analyze a clinical trial using the breast as the unit of analysis.

*Scenario 1*. Imagine a colleague has conducted a study on complications following breast reconstruction, with and without the use of an acellular dermal matrix (ADM). They provide you with a data set containing 100 observations of postoperative complications from 2 breasts each of 50 females. ADM was used for only 25 of the females. Your colleague wants to determine if using ADM has a higher incidence of complications. They analyze the sample of 100 breasts using an independent groups *t* test to compare the mean number of complications between the 2 groups. When they submit the paper to a journal, it is rejected because a reviewer points out that they are analyzing the pairs of female breasts as if they came from different females. The test is too likely to reject the possibility that there is no effect of the treatment. The correct analysis must differentiate between the variability expected among breasts of different females and the typically lower variability between 2 breasts of the same female. One such analysis is a 2-way analysis of variance with ADM (yes/no), a between-female factor, and breast (L/R) as a within-female factor.

*Scenario 2*. Now, imagine you have operated on both breasts of each patient using different methods (right side with ADM and left side without ADM), or with different exposures (1 breast radiated for cancer, and 1 side not), and want to compare the breasts of each patient with respect to complications. In this instance, you are interested in “within-patient” comparisons, with each patient as her own control. Here, a paired analysis will be more powerful because the variance of the paired differences will be smaller than variance of the unpaired means of the breasts of different females. Collecting paired data improves the power for paired comparisons and for any interactions between within-patient and between-patient factors. It can also be a good strategy to improve the power of comparisons of females who receive different treatments in a design in which 1 breast is treated and the other is a control. In such a case, the variability in outcome associated with factors common to the treated and untreated breasts is a nuisance, and it can be removed from the comparison of the treated breasts of different females, yielding a test that is more likely to detect an effect when it exists. One design that may also be considered is a within-patient crossover trial.^4^ For example, a scar cream or lymphatic drainage technique applied postoperatively in which the patient is treated with the intervention of interest for a period of time, has a washout period, and then applies the other comparative intervention. Regardless of which design is used, once the data are collected, valid inferences require that you estimate the correct variance of each treatment effect of interest. In these instances, it would not be wrong to use the breast for the unit of analysis, but the analysis must then tease out within-patient and between-patient comparisons to arrive at the correct inferences for both effects. In this case, analysis would require other methods other than an independent groups *t* test or Pearson chi-square analysis.

*Example*. Panton et al aimed to assess the efficacy of force-modulating tissue bridges to reduce mechanical tension on postoperative wounds in patients undergoing Wise pattern reduction mammaplasty.^5^ In this randomized controlled trial, 34 patients (68 breasts) were randomized to either standard care or the intervention and compared using a within-patient analysis. The unit of observation was wound healing, measured in each breast, and was analyzed at the level of the breast using *t* test analysis for the primary outcome and linear regression for the secondary outcome. Matched-pairs testing demonstrated that there was an association between the closure method and the risk of wound dehiscence (*P* = .009), with an odds ratio of 0.091 (95% CI, 0.002-0.625). In this instance, it was important that the paired nature of the data was accounted for during analysis, because statistical power would have been reduced if it was ignored.

*Question*. Based on the study design and results, which of the following statements, if any, are true?

1. If 2 breasts of the same female are similar with respect to the outcome, a paired analysis using within-patient comparison allows for fewer patients to be sampled than a between-patient design.
2. All trial participants acted as their own control, allowing for a within-patient analysis.
3. The secondary analysis did not require matched-pairs testing because only the primary analysis contributed to unit of analysis errors.
4. A crossover trial may be appropriate to analyze the breast as the unit of analysis.

*Answer*. Statements 1 and 2 are true. Three is incorrect because both the primary and secondary analysis can be prone to unit of analysis errors. In this example, the primary outcome analysis for wound healing was paired, while the secondary analysis using regression and binomial testing was not paired. This demonstrates that there are potential unit of analysis issues for the secondary analysis of risk factors. Four is incorrect because this intervention requires a closure method following surgery.

**Figure 1.**
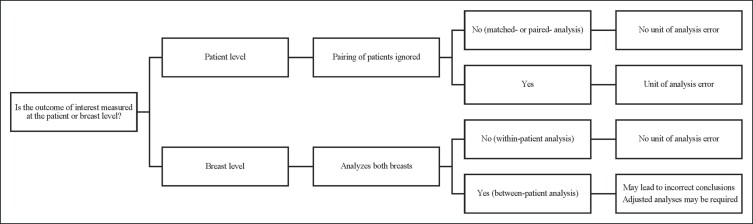
Proposed tool to determine appropriate unit of analysis for 2 symmetrical surgical sites.

In part 2 of this guide, we have demonstrated when it is appropriate to use the breast as the unit of analysis. When collecting assessments from both breasts, researchers often assume that the breasts are independent observations. The effect of ignoring pairing depends not only on the degree of similarity between the breasts of the same patient but also the design and comparison of interest. The opportunities for exploiting paired observations to improve precision also depend on whether one is interested in within-patient or between-patient comparisons. The conception of this 2-part guide arose from unit of analysis errors identified in the breast surgical literature. There continue to be challenges with reporting and conclusions drawn by authors in plastic surgery journals.^6-8^ Articles must adhere to reporting guidelines and use the appropriate analyses to inform patient care. Clinicians and researchers must critically and thoughtfully consider their unit of analysis when developing clinical studies. Many sources are available outlining how to appropriately select a unit of analysis.^9,10^ However, we have proposed a tool to guide this selection (Figure 1). Future research should aim to assess whether breast surgical studies utilize the appropriate unit of analysis.
